# Migration Capacity and Viability of Human Primary Osteoblasts in Synthetic Three-dimensional Bone Scaffolds Made of Tricalciumphosphate

**DOI:** 10.3390/ma4071249

**Published:** 2011-07-08

**Authors:** Anika Jonitz, Jan Wieding, Katrin Lochner, Matthias Cornelsen, Hermann Seitz, Doris Hansmann, Rainer Bader

**Affiliations:** 1 Department of Orthopaedics, Biomechanics and Implant Technology Research Laboratory, University of Rostock, Rostock 18107, Germany; E-Mails: jan.wieding@med.uni-rostock.de (J.W.); katrin.lochner@med.uni-rostock.de (K.L.); doris.hansmann@med.uni-rostock.de (D.H.); rainer.bader@med.uni-rostock.de (R.B.); 2 Department of Mechanical Engineering and Marine Technology, Chair of Fluid Technology and Microfluidics, University of Rostock, Rostock 18107, Germany; E-Mails: matthias.cornelsen@uni-rostock.de (M.C.); hermann.seitz@uni-rostock.de (H.S.)

**Keywords:** human primary osteoblasts, tricalciumphosphat, scaffold, hypoxia, acidification, microsensors

## Abstract

In current therapeutic strategies, bone defects are filled up by bone auto- or allografts. Since they are limited by insufficient availability and donor site morbidity, it is necessary to find an appropriate alternative of synthetic porous bone materials. Because of their osteoconductive characteristics, ceramic materials like tricalciumphosphate (TCP) are suitable to fill up bone defects. Another advantage of TCP implants is the ability of patient-specific engineering. Objective of the present *in-vitro* study was to analyze the migration capacity and viability of human primary osteoblasts in porous three-dimensional TCP scaffolds in a static cell culture. To obtain data of the cellular supply with nutrients and oxygen, we determined the oxygen concentration and the pH value within the 3D scaffold compared to the surrounding medium using microsensors. After eight days of cultivation we found cells on all four planes. During incubation, the oxygen concentration within the scaffold decreased by approximately 8%. Furthermore, we could not demonstrate an increasing acidification in the core of the TCP scaffold. Our results suggest that osteoblasts could migrate and survive within the macroporous TCP scaffolds. The selected size of the macropores prevents overgrowth of cells, whereby the oxygen and nutrients supply is sufficiently guaranteed.

## 1. Introduction

Large-size bone defects can be the consequence of tumor, trauma or infections. Every year, there are more than 1.5 billion bone-grafting procedures worldwide [1]. In the past as the “Golden Standard”, defects were filled up by bone auto- or allografts [2,3]. Disadvantages of these therapeutic strategies are limited availability, risk of infections and donor-site morbidity [4,5]. Therefore, it is necessary to apply appropriate synthetic, porous bone substitutes [6] which can be adapted to the bone defect. These three-dimensional (3D) scaffolds should mimic the artificial extracellular matrix (ECM) so that cells are able to migrate, proliferate and differentiate within these structures [7]. However, with increasing size of synthetic scaffolds, there are increasing gradients in tissue quality in terms of inhomogeneous cell proliferation and differentiation from the periphery to the core [8,9].

Within a 3D scaffold, cells depend on diffusion processes in order to be sufficiently supplied with oxygen and nutrients as well as to remove metabolic waste [10]. In living tissue oxygen supply, nutrients and also waste elimination must be kept constant over 20 to 200 µm [10,11], whereby cells are supplied by blood vessels. Diffusion processes *in-vitro* are limited to approximately 200 µm [10]. As a consequence, gradients in environmental conditions predominate between the periphery and the core of tissue-engineered constructs [10]. Therefore, structures or channels for vascularisation within large-scaled synthetic 3D scaffolds have to be considered.

There are many synthetic, porous materials available on the market but usually they show simple geometries such as blocks or pins. Three-dimensional printing (3D printing) is a well-suited technique for generating complex porous matrices directly from powder material [12]. Anatomical information of the patients can be used for the design of individual implants for defect-filling [12,13]. Thereby, the outer shape of the implant can be closely adapted to the bone defect as well as the inner channels are optimized for cell ingrowth [14,15]. Tricalciumphosphate (TCP) is a suitable material for 3D printing. Scaffolds can be built up layer by layer with subsequent sintering [13]. Consequently, the final scaffold is highly defined with precise dimensions and regular characteristics such as the pore size [16,17]. Besides the possibility of individual design, TCP is distinguished by its osteoconductive characteristics [18,19].

However, no data are shown about the oxygen supply and pH monitoring within a 3D TCP scaffold settled by human osteoblasts so far. In the present study, we designed an experimental setup to measure the oxygen content as well as acidification in the core of the TCP scaffold compared to the surrounding medium. Therefore, we placed two TCP discs on top of each other to obtain a 3D construct with a total height of 10 mm and four planes (superior, two intermediate and inferior). We seeded human osteoblasts on the superior plane and analyzed the migration capacity of cells within the scaffold. To collect information of the oxygen content and acidification, we used optical microsensors for measuring the oxygen concentration and pH value. Furthermore, we determined the ability of type I pro-collagen synthesis of the cells.

## 2. Materials and Methods

### 2.1. Isolation and Cultivation of Human Primary Osteoblasts

Human primary osteoblasts were isolated under sterile conditions from bone marrow derived from femoral heads of patients undergoing primary total hip replacement. All samples were collected after patient’s agreement and approval by the Local Ethical Committee (registration number: A 2010-10).

Cancellous bone specimens were carved from the inside of femoral heads, washed three times with PBS (PAA, Cölbe, Germany), cut into small pieces and treated with Dulbecco´s modified Eagle medium (DMEM, Gibco^®^ Invitrogen, Darmstadt, Germany) containing collagenase A and dispase (both: Roche, Mannheim, Germany) at a ratio of 1:2:1 at 37 °C for 3 hours. Afterwards, the cell suspension was filtered through a cell strainer (pore size: 70 µm; Nunc, Wiesbaden, Germany) and centrifuged at 118 × g for 10 min. The remaining cell pellet was resuspended in complete medium containing 10% FCS, 1% Amphotericin B, 1% Penicillin-Streptomycin and 1% Hepes-Buffer (all: Gibco^®^ Invitrogen, Darmstadt, Germany).

Freshly isolated osteoblasts from 2 to 3 g wet weight were plated in a 25 cm^2^ culture flask with 8 mL complete medium mentioned above containing ascorbic acid (final concentration: 50 µg/mL), β-glycerophosphate (final concentration: 10 mM) as well as dexamethasone (final concentration: 100 mM) (all: Sigma, Seelze, Germany). Cells were incubated in a humidified atmosphere of 5% CO_2_ and 37 °C. The cell culture medium was changed every second day to remove non-adherent cells. Cell proliferation was monitored microscopically. As cells reached 90% confluency, they were trypsinized and splitted at a ratio 1:6. To verify the osteogenic character of isolated cells, alkaline phosphatase stainings were carried out.

### 2.2. Cell Settlement on Tricalciumphosphate (TCP) Scaffolds

**Figure 1:**
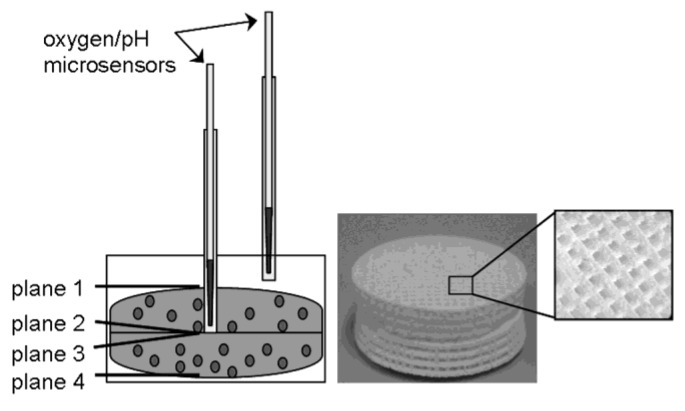
Schematic design of the 3D-TCP scaffold which consisted of two TCP discs (30 × 10 mm in size with 700 × 700 µm macropores) and therefore four different planes (plane 1: superior; plane 2 and 3: intermediate, plane 4: inferior).

Human osteoblasts were seeded on porous cylindrical TCP scaffolds which were fabricated in a 3D printing process with loose powder and a polymer-based binder solution for local initial bonding of the powder. The CaP-based granulate TCP 4 was obtained from BioCer Entwicklungs-GmbH (Bayreuth, Germany). The printed ceramic green bodies were consolidated at a temperature of 1250 °C to improve their mechanical properties [12]. The disc-shaped TCP constructs were 30 × 5 mm in size with macropores of 700 × 700 µm in all three spatial directions. To distinguish between different depths, the complete scaffold module was composed of two TCP discs which were placed on top of each other (Figure 1). In addition, the upper TCP disc had a central hole to insert a standard hollow needle for oxygen and pH measurement (see 2.3. and 2.4.).

Before cell seeding, the TCP scaffolds were incubated in complete medium under standard culture conditions for 72 hours. Subsequently, 4 × 10^5^ cells were seeded on the superior plane by dropping a 10 µL cell suspension point-wise on the scaffold. After 45 minutes, complete medium was refilled and the construct was incubated at 37 °C and 5% CO_2_ for eight days. Medium was changed completely every second day.

### 2.3. Oxygen Measurements

For the oxygen measurements, needle-type oxygen microsensors (Oxygen Micro-Optode, Type PSt1; Presens, Regensburg, Germany), mounted on optic fibers with a 140 µm tip, were used. For protection of the fragile sensors, they were fixed within a standard hollow needle of 0.4 mm diameter. We used standard hollow needles with a diameter of 1.02 mm to achieve higher mechanical stability during measurements (Figure 1). A two-point calibration was performed before the measurements using oxygen-free water (0% air saturation) and water vapor-saturated air (100% air saturation). Oxygen consumption was measured both in the core and the periphery of the 3D scaffold every 24 h over a period of 30 minutes for eight days.

### 2.4. pH Monitoring

To monitor the pH values within the 3D-cell culture, needle-type pH microsensors (pH microsensor; Presens, Regensburg, Germany), mounted on optic fibers with a tip of less than 150 µm, were used. For protection of the fragile sensors, they were fixed within a standard hollow needle of 0.4 mm diameter. Analogue to the oxygen measurements we used standard hollow needles with a diameter of 1.02 mm to improve the stability during pH measurements (Figure 1). Prior to the measurement a calibration with buffer solutions of pH 4 to 7 (all: Roth, Karlsruhe, Germany) was performed. The pH value was monitored both in the periphery and in the core of the 3D scaffold every 24 h over a period of 30 min for eight days.

### 2.5. WST-1 Assay

The mitochondrial activity of osteoblasts on the TCP scaffold was analyzed by using a WST-1 assay (Roche, Mannheim, Germany). The background of this assay is, that cells with a high metabolism cleave the tetrazolium salt WST-1 to formazan in direct dependency on their metabolic activity [20]. Therefore, the TCP module was covered with assay reagent (ratio between complete medium to WST-1 reagent of 10:1). Additionally, a medium control was used. After an incubation time of 60 minutes under cell culture conditions, 200 µL aliquots of assay reagent were transferred to a 96-well format. Absorbance was measured at 450 nm (reference 630 nm) using an Opsys MR™ microplate reader (Dynex Technologies, Denkendorf, Germany).

### 2.6. Pro-Collagen Type I Quantification

Synthesis of pro-collagen type I (Metra™ CICP EIA Kit, Quidel, Marburg, Germany) was determined by an enzyme-linked immunosorbent assay (ELISA). For the analysis, 500 µL medium were removed from the core and the periphery of the TCP scaffold. The assay was performed according to the manufacturer’s instructions. Absorbance was measured at 405 nm using the Opsys MR™ microplate reader (Dynex Technologies, Denkendorf, Germany).

### 2.7. LIVE/DEAD^®^ Assay

By means of the LIVE/DEAD^®^ viability/cytotoxicity kit (Invitrogen, Darmstadt, Germany), cell viability was determined. Hence, both TCP discs were incubated in LIVE/DEAD^®^ assay reagent containing calcein AM and ethidium homodimer. Calcein AM is the acetoxymethyl ester derivative of the fluorescent dye calcein. Calcein AM is membrane-permeant and thus is taken up by cells incubated with PBS supplemented with calcein AM. Once inside the cells, calcein AM is hydrolyzed by endogenous esterases into the polyanionic non-membrane permeant green fluorescent dye calcein. As a result, viable cells with intact membranes produce an intense uniform green fluorescence (ex/em 495 nm/515 nm). Ethidium enters cells with damaged membranes and produces a bright red fluorescence (ex/em 495 nm/635 nm) to indicate dead cells. After 30 min of incubation at room temperature with PBS containing both dyes, images of the cells were taken using a fluorescence microscope with a fourfold magnification object lens (Nikon ECLIPSE TS100; Nikon GmbH, Düsseldorf, Germany).

### 2.8. Statistical Analysis

Data are presented as mean values ± standard deviation. Statistical significance between groups was calculated by an ONEWAY ANOVA (Post Hoc LSD) test, the Mann-Whitney-U-Test or the student`s t-Test using SPSS 15.0 for Windows (SPSS Inc., Chicago, IL, USA). A value of p < 0.05 was considered significant. A minimum of three independent experimental runs were performed for all analysis.

## 3. Results

### 3.1. Migration Capacity and Viability of Human Osteoblasts Within the TCP Scaffold Module

**Figure 2:**
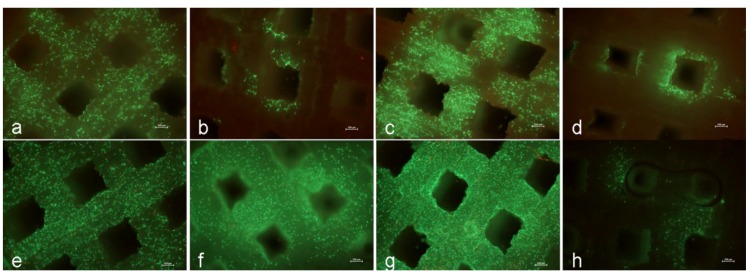
(**a**–**h**): Migration capacity and viability of human primary osteoblasts on the different planes of the 3D TCP scaffold on day one (**a**–**d**) and after eight days (**e**–**h**) of cultivation under static culture conditions (n ≥ 3; living cells = green, dead cells = red; bar: 250 µm); **a**, **e**: plane 1; **b**, **f**: plane 2; **c**, **g**: plane 3; **d**, **h**: plane 4.

To analyze the migration potential of human primary osteoblasts, cells were seeded on the superior plane 1. One day after seeding, a lot of cells were observed on plane 1 and 3, whereas only few cells were detected on plane 2 and 4. On day 8, a lot of viable cells could be detected on planes 1 to 3. These cells also formed numerous filopodia for cell connections. In contrast, only few cells were on inferior plane 4. Furthermore, the number of dead cells, especially on plane 1 and 3, slightly increased (Figure 2).

**Figure 3:**
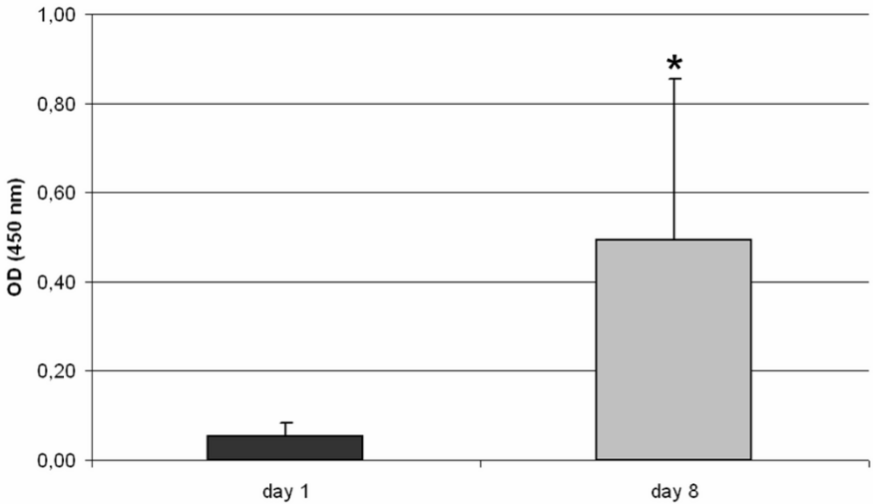
Metabolic activity of human osteoblasts seeded on the 3D TCP scaffold after eight days of cultivation (n ≥ 3). Data are presented as mean value ± standard deviation. Statistical analysis was done with a Mann-Whitney-U-Test. Significance is based on the OD of day 1 (* p < 0.05).

After one and eight days of cultivation, the experimental setup was disassembled in order to carry out the WST-1 assay. On both time points, we could detect metabolically active cells, whereby the metabolic activity significantly (p = 0.025) increased ten-fold during the incubation time (Figure 3).

### 3.2. Monitoring of Oxygen Consumption and Acidification

In order to obtain details of the supply of oxygen within the core, the oxygen content was determined every day. For comparison, the content of oxygen was also measured in the surrounding medium of the scaffold.

**Figure 4:**
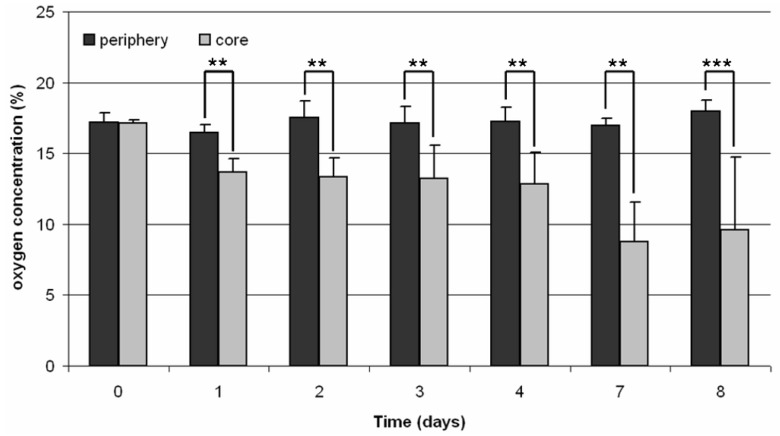
Oxygen concentration in a static 3D cell culture of human osteoblasts (n = 4). Measurement was done in the periphery as well as in the core of the TCP scaffold. Data are demonstrated as mean values ± standard deviation. Statistical analysis was done with ONEWAY ANOVA (Post hoc LSD). Significance is based on the respective oxygen concentration of the periphery (** p < 0.01; *** p < 0.001).

After cell seeding (day 1), there was no difference between the oxygen content in the core and the periphery of the TCP module. In contrast, from day two to day eight a reduction in oxygen level within the scaffold compared to the surrounding medium was detected. During the incubation the oxygen content in the core decreased significantly from 17.2% (day 1) to 9.6% (day 8; Figure 4).

**Figure 5:**
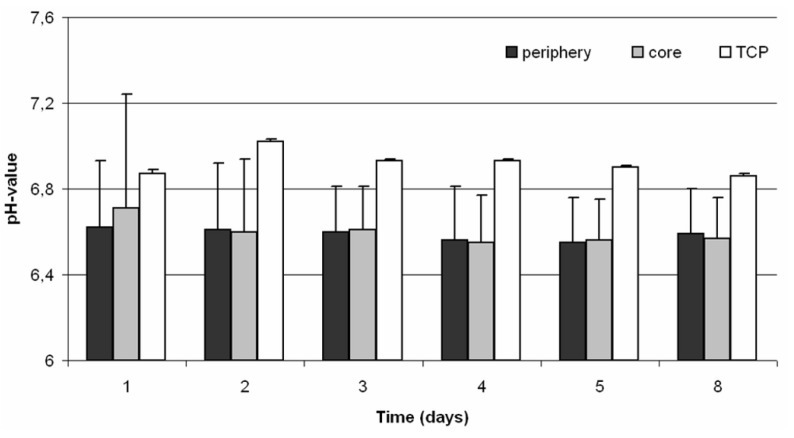
Monitoring of the pH value in a static 3D culture of human osteoblasts (n ≥ 3). Measurement was done in the core and in the periphery of the TCP scaffold over a period of eight days. Data are demonstrated as mean values ± standard deviation. Statistical analysis was done with ONEWAY ANOVA (Post hoc LSD). Significance is based on the respective pH value of the periphery.

Monitoring of the pH value showed no significant differences in acidification between the core of the scaffold and the surrounding medium during incubation. However, an acidification of the cell culture medium after cell seeding compared to the unsettled TCP scaffold was assessed (Figure 5).

### 3.3. Pro-Collagen Type I Synthesis

**Figure 6:**
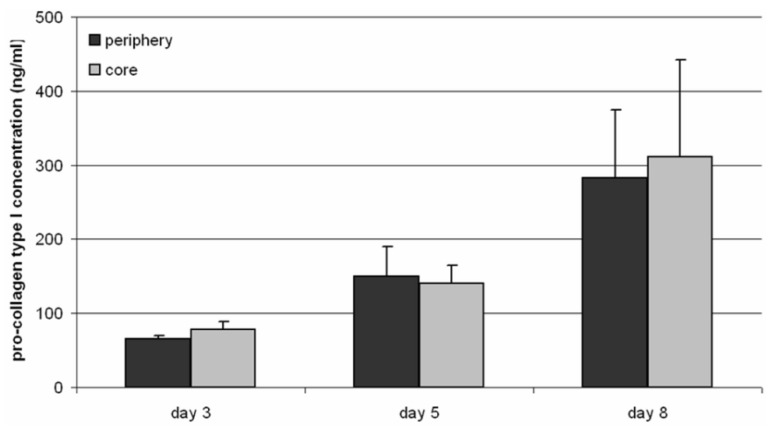
Synthesis of pro-collagen type I of human osteoblasts seeded on the 3D TCP scaffold. Supernatants were collected after 3, 5 and 8 days of cultivation and analyzed by ELISA (n = 4). Data are presented as mean values ± standard deviation. Statistical analysis was done with the student’s t-test.

To gain information on the synthesis of ECM components, the medium of the core as well as the periphery was analyzed after 3, 5 and 8 days of cultivation. The synthesis rate of type I pro-collagen increased from 65 ng/mL to 283 ng/mL in the periphery and from 78 ng/mL to 311 ng/mL within the core over the period of time, but without a statistically significant difference between the periphery and the core (Figure 6).

## 4. Discussion

For the treatment of large bone defects, the availability of suitable substitutes, e.g., bone auto- or allografts, is limited. Therefore, alternatives such as synthetic, porous 3D scaffolds are becoming more relevant. An important aspect for the design of such constructs is the individual production of implants which was achieved by 3D printing processes [12]. A suitable material for this application is TCP.

The objective of this study was to examine the consumption of oxygen as well as the acidification of the cell culture medium after settlement of human osteoblasts within a 3D TCP scaffold. Furthermore, the migration capacity and viability of cells was determined.

In contrast to Warnke *et al*. [13] we could show, that human osteoblasts were able to survive on the TCP construct. After one day of incubation, a lot of viable cells were detected on the surface of the scaffold which was in accordance to Becker *et al.* [19]. During incubation, cells migrated through the construct, so that the scaffold was almost fully occupied by cells. Furthermore, verification of the metabolic activity revealed a significant increase of metabolic activity by osteoblasts from day one to day eight.

An important aspect for the design of large-scaled scaffolds is the sufficient supply of oxygen and nutrients within the constructs. In engineered constructs, the transport of oxygen and nutrients is only maintained by diffusion processes [10]. Therefore, gradients in environmental conditions predominate between the periphery and the core of 3D scaffolds [10].

Monitoring of the oxygen concentration within the TCP scaffold showed significant differences between the core and periphery. The difference of the oxygen concentration within the scaffold compared to the surrounding medium was approximately 4% during the first five days of incubation. This difference increased further to approximately 8% on the last day of cultivation so that the remaining oxygen concentration within the scaffold was at 9.6%. This was in contrast to Volkmer *et al*. [9] where a decrease of the oxygen concentration to 0% was detected for the scaffolds used after five days of incubation.

Furthermore, the acidification of the cell culture medium within and around the TCP scaffold was monitored. It is well described that hypoxia is associated with a local decrease of the pH value which is caused by an anaerobic cell metabolism [21]. In addition, small pH value reductions could influence the matrix deposition by osteoblasts negatively [21]. The results showed that there were no differences between the periphery and the core of the cell-seeded scaffold, although a difference in the pH value of approximately 0.3 between the cell-seeded and untreated scaffold was demonstrated. Nevertheless, the pH value of the untreated TCP scaffold was about 6.9, which demonstrates that TCP is a material with acidic characteristics.

Hypoxia as well as acidic pH values could influence matrix deposition by cells [21,22]. In our experiments an increasing type I pro-collagen synthesis rate during cultivation was determined, thus collagen expression was unaffected by lower pH values and hypoxia.

Our results suggest that human osteoblasts could survive on porous TCP scaffolds in a static cell culture. Furthermore, cells migrated through the macropores to the inner structures of the scaffold. Hereby, the synthesis of pro-collagen type I was sustained during the incubation time and evidently increased. Hence, matrix production and therefore overgrowing of pores by matrix deposition, which was reported by Malda *et al*. [10], can influence and hinder the diffusion of oxygen slightly within the 3D construct. The macropores of the TCP scaffold were settled by cells but the macropore size prevented an overgrowing of cells. Thus, the used pore-size had a positive influence of cell survival and the supply of oxygen and nutrients was sufficiently guaranteed in the static cell culture. Furthermore, the monitoring of cell migration, oxygen consumption as well as acidification could be a helpful instrument for creating 3D bone implants enabling a sufficient ingrowth of human bone cells. Nevertheless, in further studies it is recommended to modify the *in-vitro* conditions towards more physiological conditions. Therefore, it is necessary to analyze cell viability on 3D scaffolds in a dynamic cell culture. Moreover, vascularisation within the scaffold has to be performed to guarantee a sufficient oxygen and nutrient supply.
